# An improved segmentation algorithm for quantification of myocardial infarction in contrast enhanced CMR images - validated in ex-vivo studies

**DOI:** 10.1186/1532-429X-16-S1-P360

**Published:** 2014-01-16

**Authors:** Felicia Seemann, Jane Tufvesson, Robert Jablonowski, Henrik Engblom, Sasha Koul, Jesper van der Pals, Håkan Arheden, Einar Heiberg

**Affiliations:** 1 Department of Clinical Physiology, Institution for Clinical Sciences, Lund University, Lund, Sweden; 2 Centre for Mathematical Sciences, Lund University, Lund, Sweden; 3 Department of Cardiology, Institution for Clinical Sciences, Lund University, Lund, Sweden

## Background

T1-weighted contrast enhanced CMR images of explanted hearts in experimental studies provide a high resolution reference standard for quantification of myocardial infarction. Manual delineation is considered the reference standard, however it is time consuming and observer dependent. Especially since a data set may consist of more than 150 images with a resolution of typically 0.5×0.5×0.5 mm. Previous studies have used an algorithm for infarct quantification based on a fix number of 8 standard deviations (SD) from remote with manual corrections if necessary [1]. This algorithm may fail if images have different contrast to noise ratio, and is time consuming as the user needs to draw regions of interest in each slice. Therefore, the aim of this study was to provide a fully automated segmentation algorithm for quantification of myocardial infarction in T1-weighted contrast enhanced high resolution ex-vivo images.

## Methods

The study included 18 explanted hearts from pigs with experimentally induced infarction by occlusion of the left anterior descending artery. Segmentations by the SD-algorithm with manual corrections were used as reference standard. The proposed algorithm uses a k-means algorithm to detect whether infarction is present or not in each slice. The distinction between scar and remote normal tissue is calculated with an Expectation-Maximization (EM) algorithm, followed by inclusion of microvascular obstruction into the scar area, if present. The algorithm was evaluated as the difference in scar volume between the new proposed algorithm and the reference standard. The difference in scar volume was also calculated between the SD algorithm without manual correction and the reference standard for comparison. Differences are presented as mean ± SD in percent of left ventricular mass (LVM).

## Results

**Figure 1:**
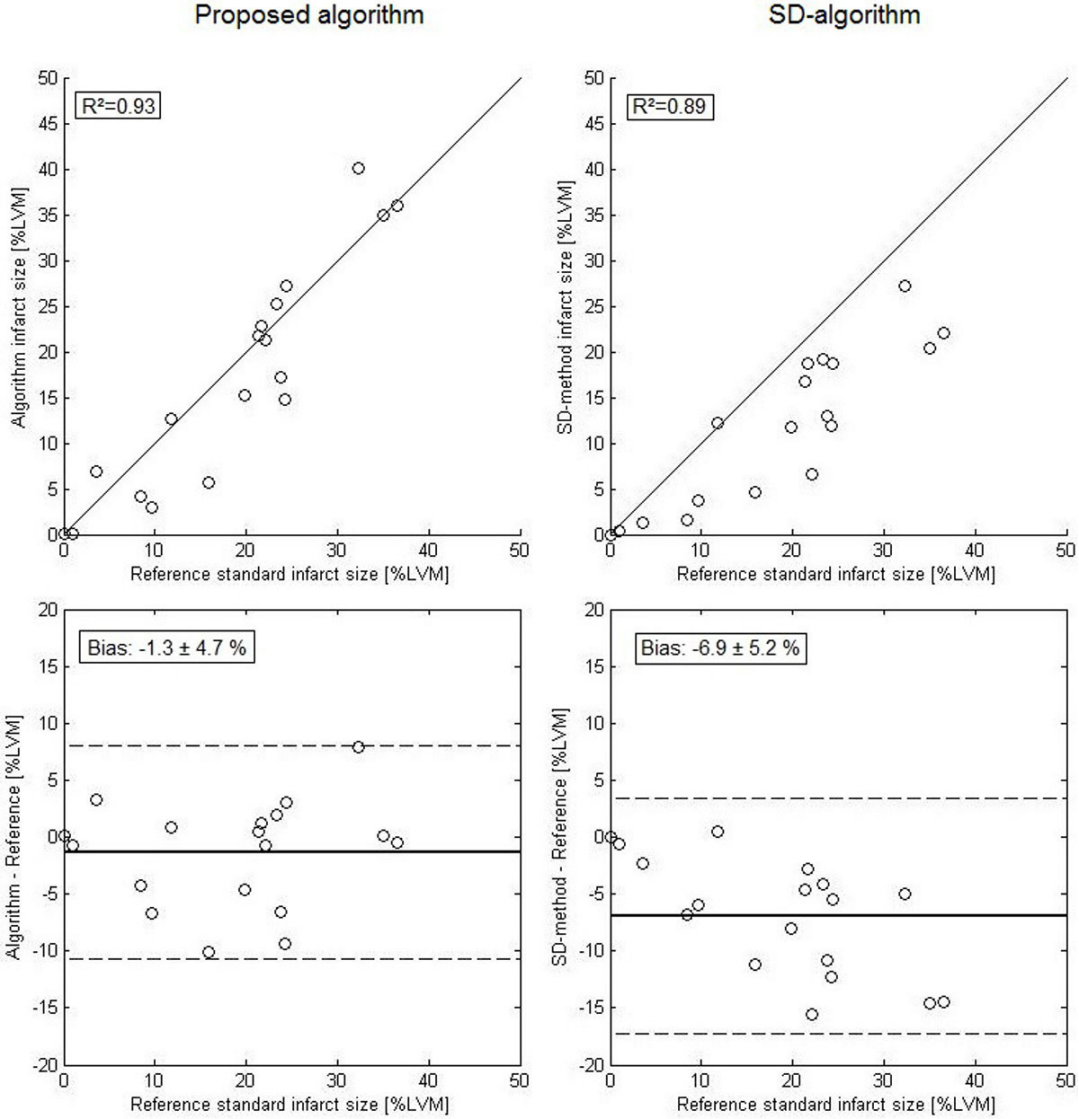
**Top row show correlations between automatic algorithms and the reference standard**. Bottom row show the difference between the algorithms and reference infarct size. Left column shows the proposed algorithm and right column shows the SD-algorithm.

**Figure 2:**
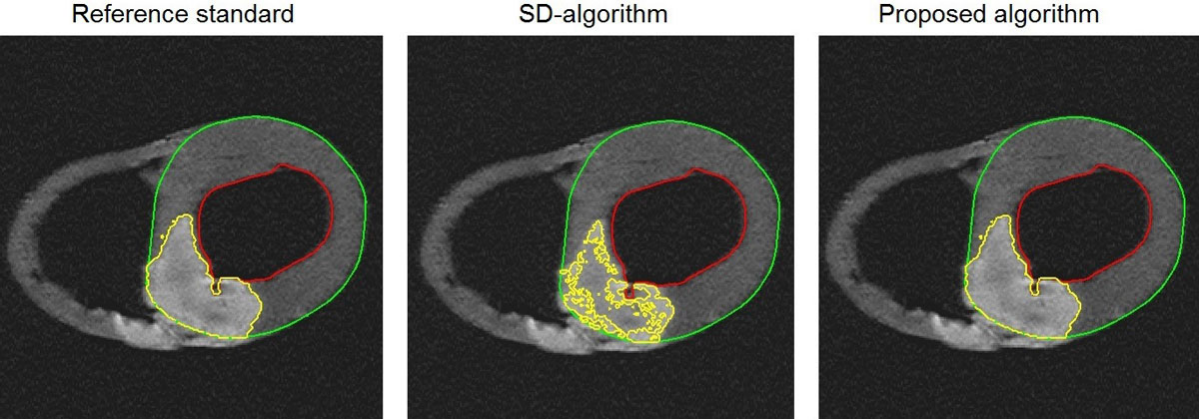
**An example of infarct segmentations by the two automatic algorithms compared to the reference standard**.

The difference between the proposed automatic algorithm and the reference standard was -1.3 ± 4.7% of LVM (R^2 ^= 0.93). The difference between the SD-algorithm and the reference standard was -6.9 ± 5.2% of LVM (R^2 ^= 0.89) (Figure 1). Figure 2 shows an example of an infarct segmentation of the two segmentation algorithms and the reference standard. The difference between the proposed algorithm and the reference standard was not statistically significant. However, the difference between the SD-algorithm and the reference standard was significant, p = < 0.01.

## Conclusions

The proposed automatic segmentation algorithm for quantification of myocardial infarction shows good agreement and low bias with the reference standard. The algorithm show potential for fully automatic quantification of myocardial infarction in high resolution contrast enhanced ex-vivo images.

## Funding

Swedish Research Council, Region of Scania.
